# Structural, electronic and photovoltaic characterization of multiwalled carbon nanotubes grown directly on stainless steel

**DOI:** 10.3762/bjnano.3.42

**Published:** 2012-05-02

**Authors:** Luca Camilli, Manuela Scarselli, Silvano Del Gobbo, Paola Castrucci, Eric Gautron, Maurizio De Crescenzi

**Affiliations:** 1 Dipartimento di Fisica, Università di Roma Tor Vergata, 00133 Roma, Italy; 2 Institut des Matériaux Jean Rouxel, CNRS-UMR 6502, 44322 Nantes Cedex 3, France

**Keywords:** carbon nanotubes, electronic properties, heterojunction, photovoltaic, stainless steel

## Abstract

We have taken advantage of the native surface roughness and the iron content of AISI-316 stainless steel to grow multiwalled carbon nanotubes (MWCNTs) by chemical vapour deposition without the addition of an external catalyst. The structural and electronic properties of the synthesized carbon nanostructures have been investigated by a range of electron microscopy and spectroscopy techniques. The results show the good quality and the high graphitization degree of the synthesized MWCNTs. Through energy-loss spectroscopy we found that the electronic properties of these nanostructures are markedly different from those of highly oriented pyrolytic graphite (HOPG). Notably, a broadening of the π-plasmon peak in the case of MWCNTs is evident. In addition, a photocurrent was measured when MWCNTs were airbrushed onto a silicon substrate. External quantum efficiency (EQE) and photocurrent values were reported both in planar and in top-down geometry of the device. Marked differences in the line shapes and intensities were found for the two configurations, suggesting that two different mechanisms of photocurrent generation and charge collection are in operation. From this comparison, we are able to conclude that the silicon substrate plays an important role in the production of electron–hole pairs.

## Introduction

Carbon nanotubes (CNTs) possess unique electronic, mechanical and optical properties that make them interesting for fundamental studies as well as practical applications [1]. Among the various synthesis techniques, chemical vapour deposition is preferred in the field of electronics, since it allows for the direct growth of CNTs on substrates [2]. CNTs are generally synthesized on Si or Si/SiO_2_ substrates; however, in some types of applications, such as in field-emission devices, the use of conductive substrates is particularly mandatory [3]. In particular, stainless steel (SS) is one of the most attractive conductive substrates, due to its low price and good processability. Recently, several efforts have been made to grow CNTs on SS substrates with [4–5] or without [6–7] the addition of an external catalyst. In particular, we have shown that the growth of high quality multiwalled CNTs on SS in the absence of an external catalyst is possible because the nanoscale roughness and the metallic nature of the substrate surface both act as an efficient catalyst/template in the synthesis of tubular nanostructures [7]. Here we first investigate the electronic properties of CNTs with the aim to compare them to those of graphite. As is well known, multiwalled CNTs, conversely to single-walled ones, should have a structure very similar to that of graphite, due to the great number of walls and the reduced radius of curvature. Despite this, we find that the CNT electronic properties obtained by energy-loss spectroscopy (ELS) in the loss region up to 30 eV from the elastic peak are quite different to those of graphite. In particular, a broadening of the π-plasmon of CNTs is found, which can be ascribed to the presence of several structures that are completely absent in HOPG and probably due to transitions among Van Hove singularities.

Here we also show that MWCNTs exhibit an interesting photovoltaic activity when they are deposited on a crystalline silicon substrate by the airbrush method. We recall that, while several efforts have been devoted to the build-up of photovoltaic devices based on a SWCNT–Si heterojunction, achieving a surprising efficiency of up to 11% [8], just a few works reported the ability of MWCNTs to serve as an energy-conversion material [9–10]. In this paper, we build up a simple photovoltaic device based on MWCNT–Si Schottky heterojunction. Photovoltaic measurements for the in-plane and top-down geometries of the device were performed. In particular, we find that the top-down geometry gives a photocurrent intensity and an external quantum efficiency (EQE) value much higher than those measured in the in-plane configuration.

## Results and Discussion

**Figure 1:**
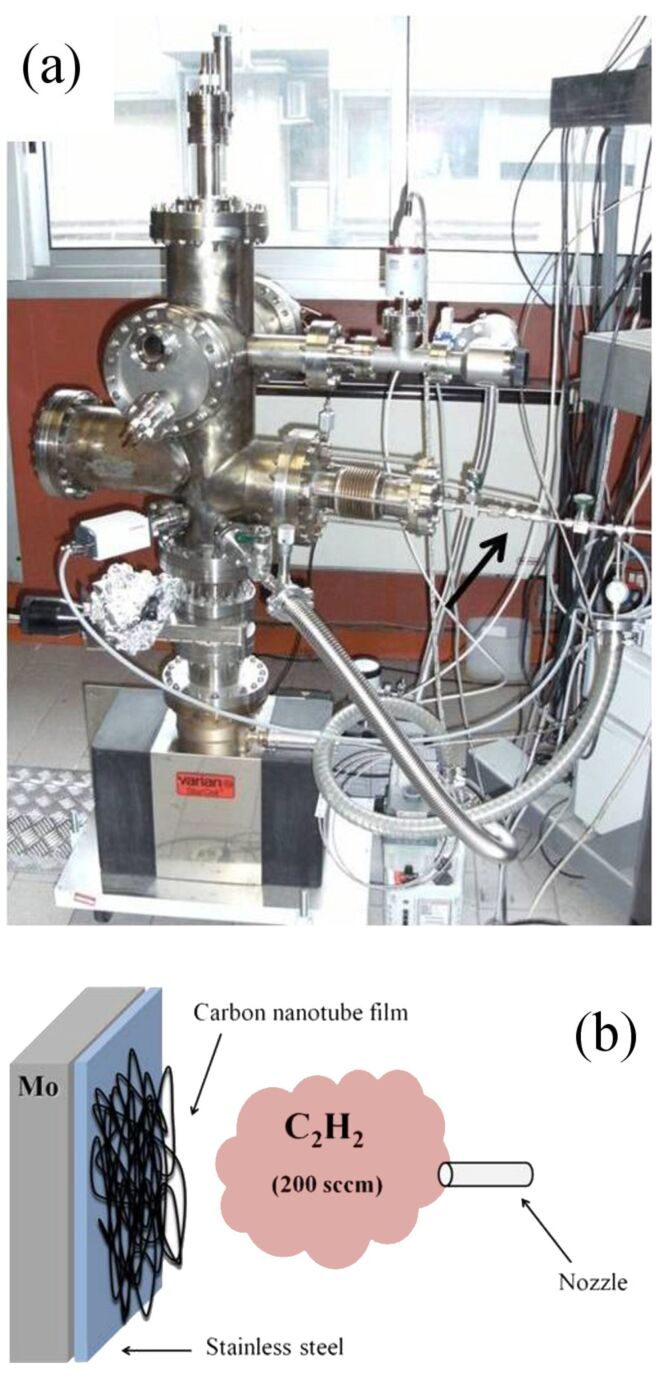
(a) Photograph of the chemical vapour deposition chamber used to synthesize MWCNTs. The reactor comprises an UHV chamber, pumped by an ion pump and equipped with a manipulator capable of reaching temperatures of about 800–900 °C. Acetylene and argon fluxes, introduced in the chamber through the gas line highlighted by the arrow, are adjusted by means of two suitable valves. (b) Schematics showing the CVD process: The stainless-steel substrate is mounted on a heatable molybdenum sample holder. After a temperature of 750 °C has been achieved, acetylene gas (200 sccm) is inserted through a nozzle in the chamber under an Ar atmosphere.

In Figure 1 the chemical vapour deposition chamber used to grow the CNTs is displayed. The stainless-steel substrate is mounted on a sample holder and heated up to 730 °C in acetylene atmosphere to synthesize CNTs (see the Experimental section for more details).

**Figure 2:**
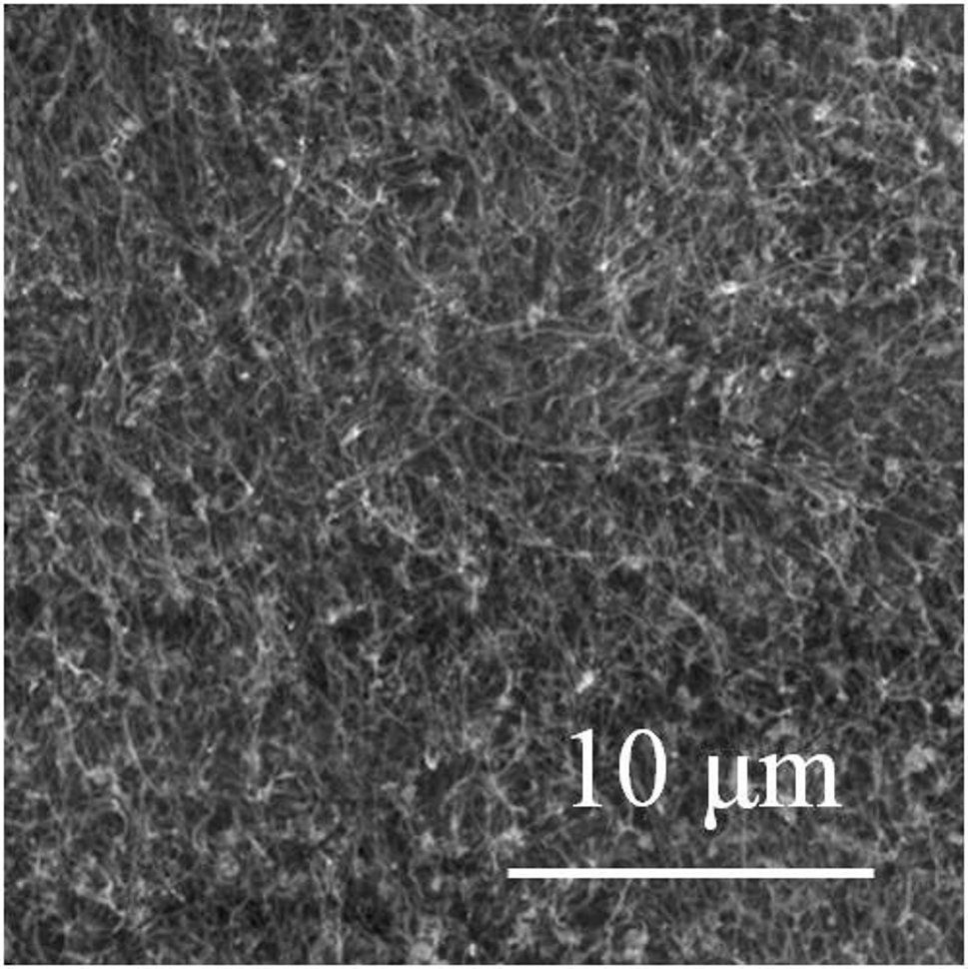
SEM image of the MWCNTs after growth on a stainless-steel substrate. The MWCNTs are randomly oriented, and no traces of other carbonaceous materials were detected in the large scanned area.

**Figure 3:**
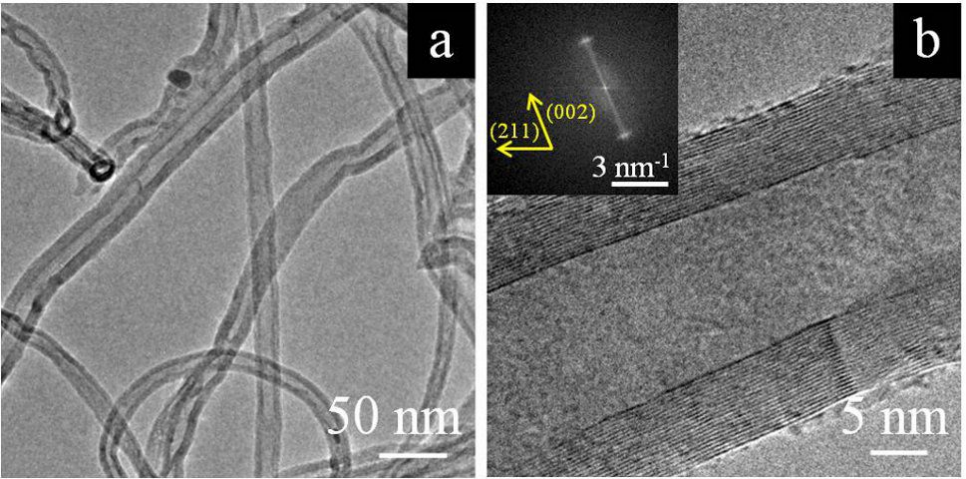
(a) Low-resolution TEM image assessing the multiwalled nature of the carbon nanotubes synthesized on SS. (b) High-resolution TEM picture of a single MWCNT with 19 walls. The inset displays the Fast Fourier Transform of the TEM image reported in (b). The two spots, at a semidistance of 0.35 nm, represent the parallel walls.

Figure 2 shows a scanning electron microscopy image of the produced CNTs. They appear to be randomly oriented and no traces of other carbonaceous materials were detected in the scanned area. In general, only a very few traces of impurities were found in other spots of the sample. Transmission electron microscopy reveals that the CNTs are multiwalled in nature, with an average number of walls of about 20, as illustrated in Figure 3a and Figure 3b. The high graphitization degree of the synthesized nanostructures is evidenced in the inset of Figure 3b, in which the reported Fast Fourier Transform of the TEM image consists of two single points located at 0.35 nm from the centre.

**Figure 4:**
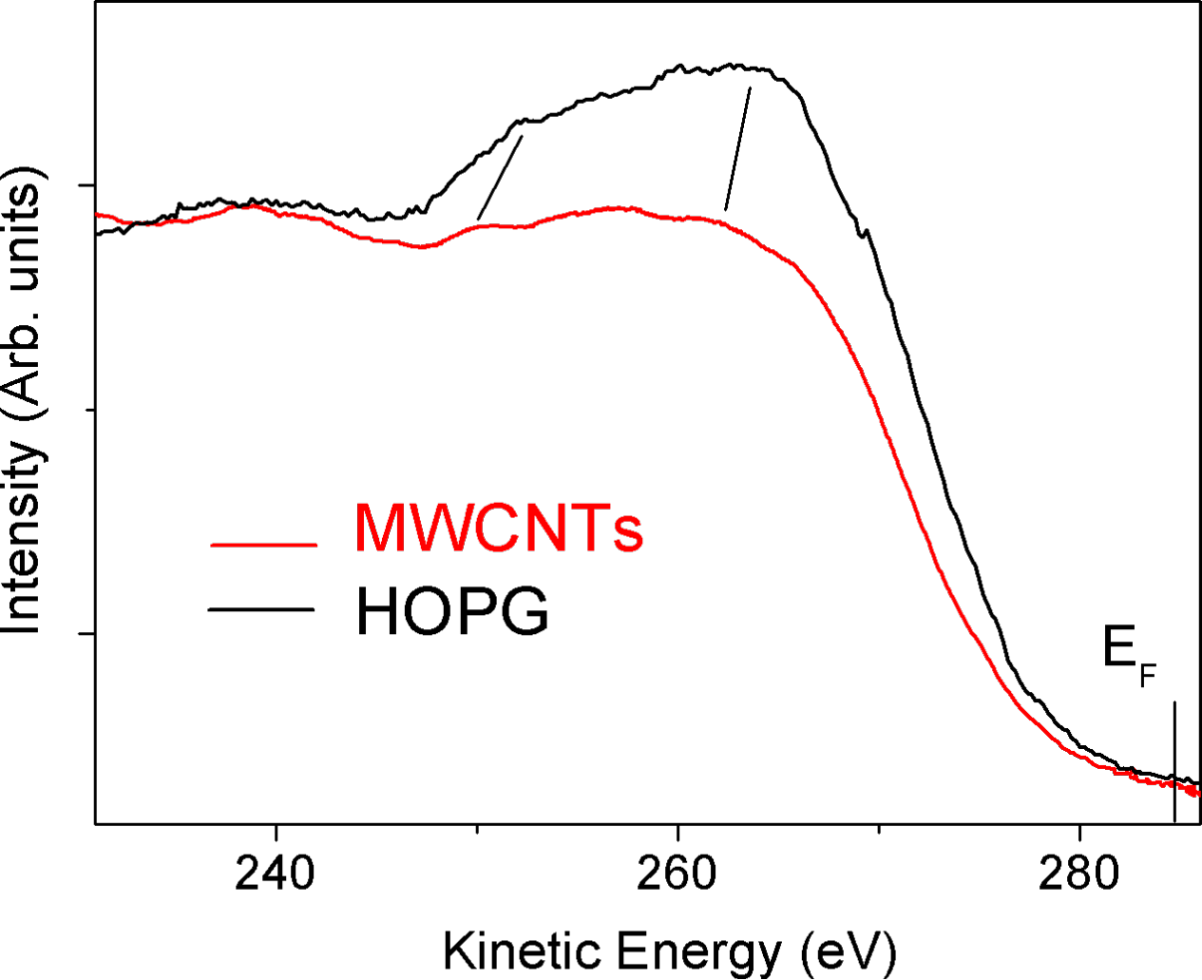
Core-valence-valence (CVV) Auger spectra of MWCNTs (red curve) and HOPG (black curve). An electron gun with a primary beam energy of 1500 eV was used as the exciting source. In the case of the MWCNT sample, the Auger peak is shifted towards lower kinetic energy with respect to HOPG. The Fermi level is at 284.4 eV.

In Figure 4 we report the carbon core-valence-valence (CVV) Auger features for HOPG and a MWCNT film. The Auger spectra were acquired using an electron gun as the exciting source with a primary beam energy of 1500 eV. Bearing in mind that the Auger spectrum is a self-convolution of the filled valence states below the Fermi level, the two experimental spectra reported in Figure 4 show some significant differences, meaning that the filled states of the two materials are intrinsically dissimilar. The shift of the peaks is highlighted by thick markers. Similar results have already been reported [11], in which the Auger line shape of HOPG was compared to that of SWCNTs.

**Figure 5:**
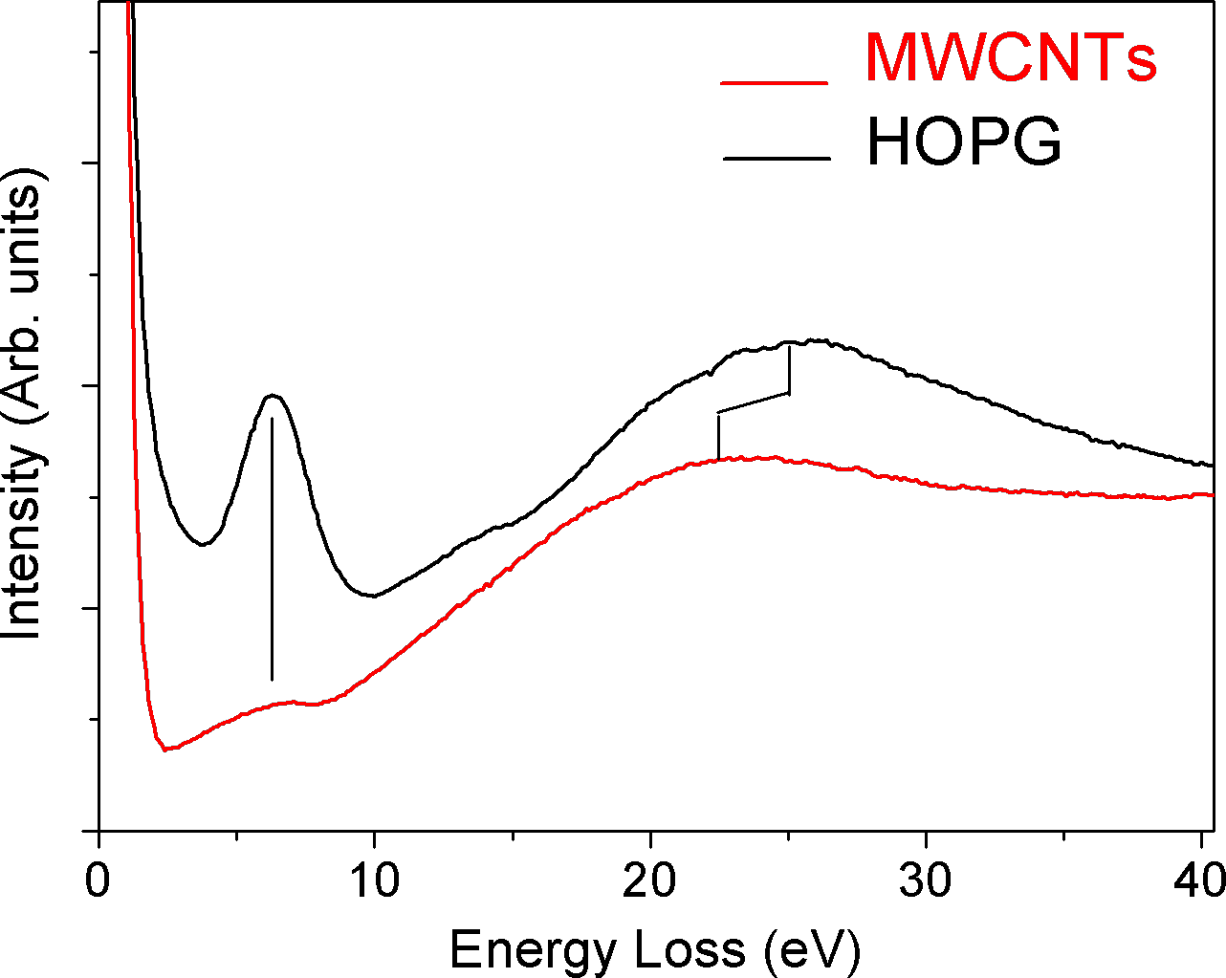
Energy-loss spectra of MWCNTs (red curve) and HOPG (black curve). In the case of MWCNTs, the σ*+*π-plasmon is shifted towards lower energy with respect to that of HOPG, located at about 26 eV, as indicated by the tick marker. The π-plasmon turns out to be sharp in HOPG (centred at 6.5 eV), whereas it is broader in MWCNTs.

Figure 5 reports the ELS analysis performed, in reflection geometry, on both MWCNT and HOPG samples. We used an electron gun as the excitation source, with a primary beam energy of 300 eV. This value has been chosen to single out the contribution coming from the first layers of the MWCNT film only. The HOPG and MWCNT spectra both exhibit two characteristic features due to the π- and σ+π-plasmons [12], although in the case of the MWCNT structures these are shifted towards lower energy with respect to those of graphite (located at 6.5 and 26 eV), indicating a marked difference in the electronic properties.

**Figure 6:**
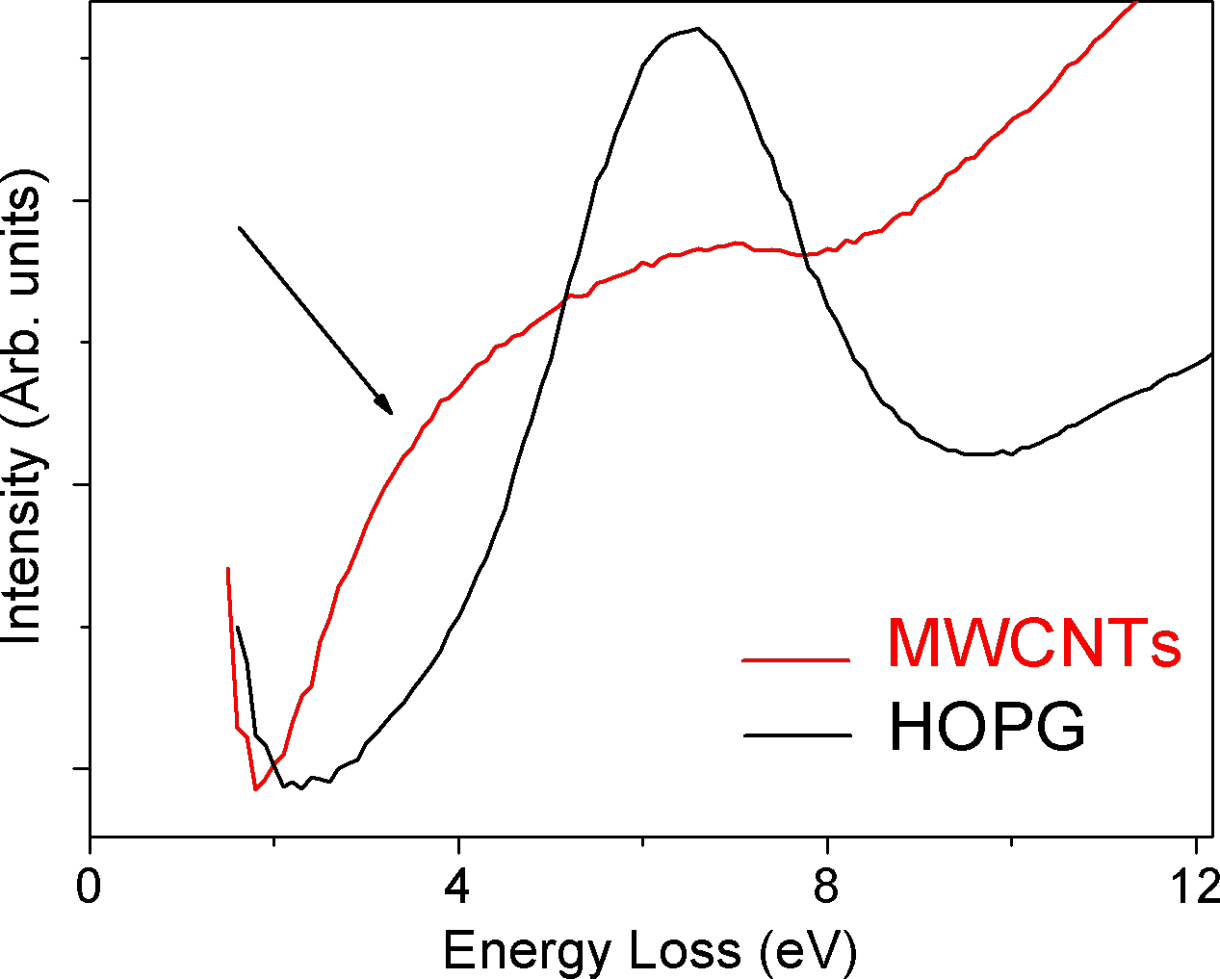
Comparison of the π-plasmon peak (0–12 eV) for MWCNTs (red curve) and HOPG (black curve). It is worth noting that in the case of MWCNTs we find electronic structures also at 2.0–4.5 eV, while in HOPG they are completely absent. These features contribute to the π*-*plasmon, giving rise to a very broad peak. Transitions between Van Hove singularities are thought to be responsible for these additional features.

Experimental evidence has shown that these plasmons exhibit an increase energetic downshift as the number of walls in the individual MWCNT being probed is reduced [13]. On the other hand, based on theoretical calculations, this effect is predicted to become greater as the number of intertube or intratube interactions decreases in bundles of SWCNTs and MWCNTs, respectively [14–16]. This finding is also in good agreement with our previous results, in which we reported ELS data collected in transmission on an isolated MWCNT [9]. In addition, it is highlighted in Figure 6 that the π-plasmon of HOPG shows a sharp peak, while the same plasmon feature in the case of MWCNTs is much broader, appearing as a shoulder that spreads over 2–4 eV in energy loss. The latter case is the result of the additional contribution coming from electronic transitions completely absent in graphite and due to the quasi-one-dimensionality of the CNTs. In the case of SWCNTs, excitations between localized electronic states related to Van Hove singularities have been invoked as an interpretation for such transitions [15]. These electronic transitions create electron–hole pairs upon illumination and, thus, have been considered to be responsible for the generation of photocurrent in SWCNTs. Since in the electronic density of states of MWCNTs such a singularity is still present [9], we think that the experimentally measured shoulder should have the same origin. Shyu and Lin report a complete calculation of these energy-loss features, dependent on the number of walls in the nanotube structure [14].

**Figure 7:**
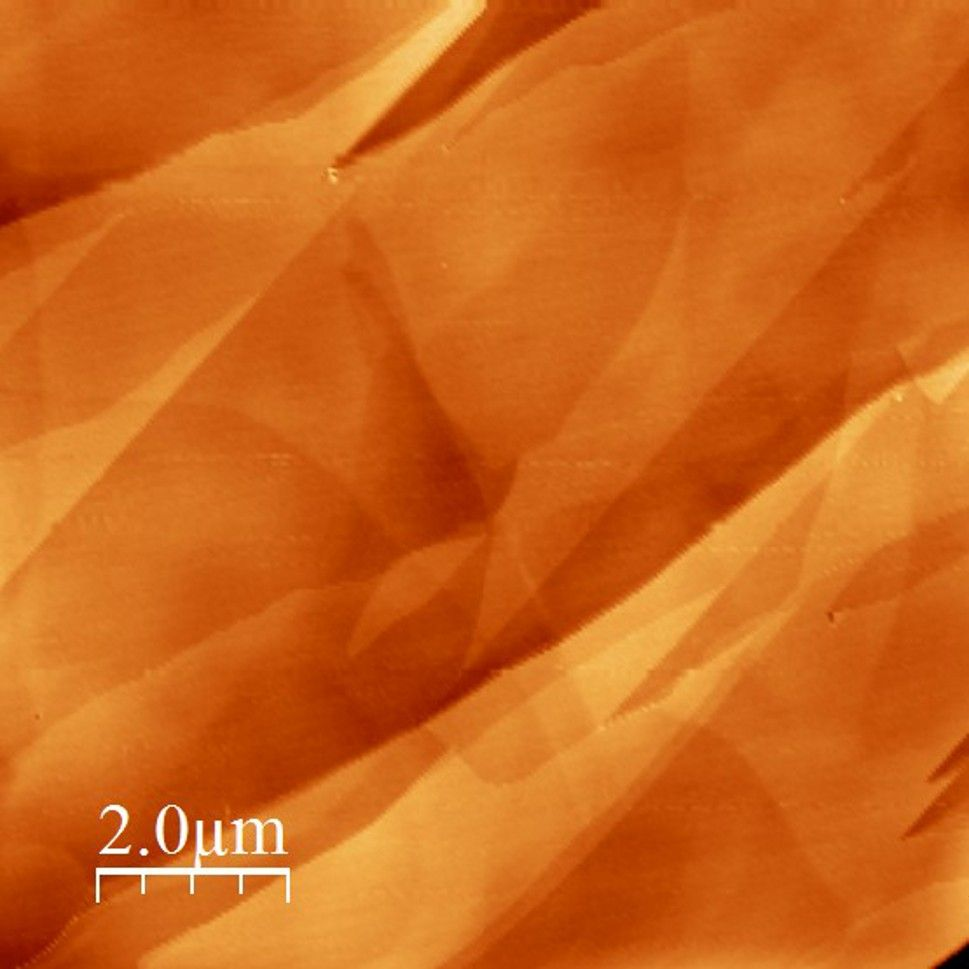
AFM 10 × 10 μm^2^ topography image of the as-exfoliated HOPG sample. The surface appears clean and several steps can be observed.

**Figure 8:**
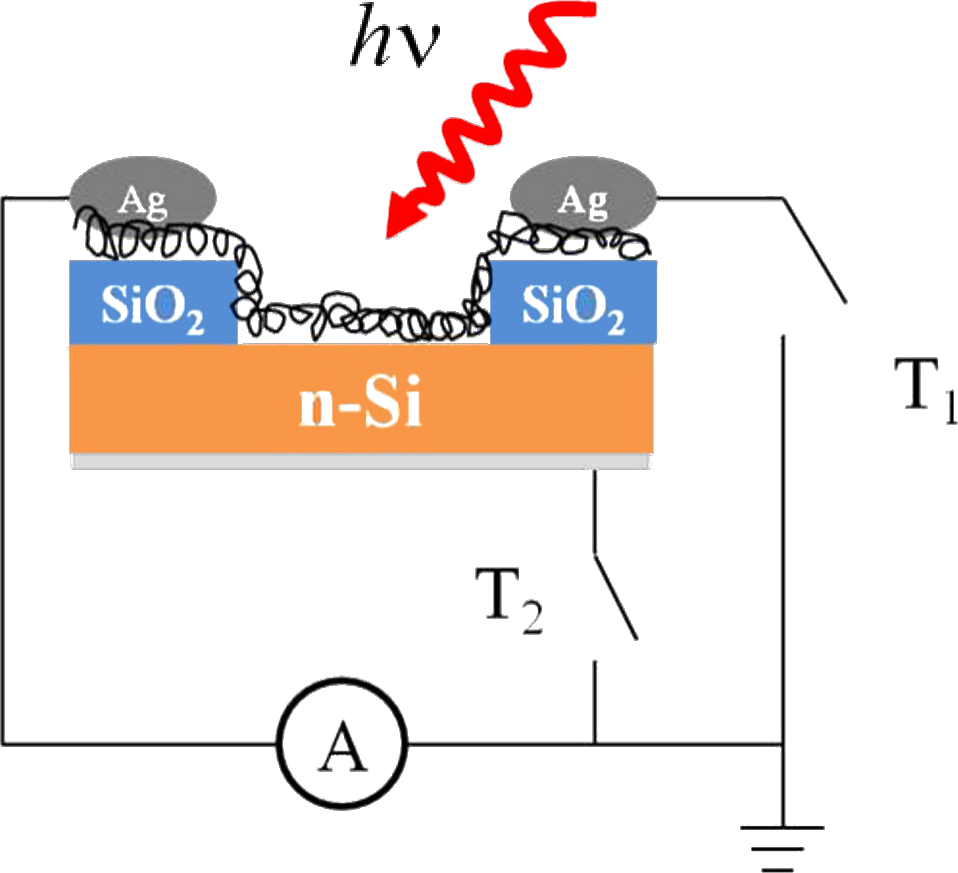
Scheme of the photovoltaic device. The Schottky junction between the Si and the MWCNT film is the photoactive junction. Steps of SiO_2_ (300 nm) are used to avoid a short-circuit forming between the silver paint (top electrode) and the silicon substrate. The back contact is made of aluminium. In the in-plane geometry, the switch T1 is on and T2 is off; and vice versa in the top-down configuration. As a result, the photocurrent is collected at the MWCNT film in the former case, and at the MWCNTs and Si (back contact) in the latter.

Figure 7 shows a 10 × 10 μm^2^ atomic force microscopy (AFM) image of the as-exfoliated HOPG sample used for spectroscopic characterization. Figure 8 shows the scheme of the two different architectures used to build and test the photovoltaic device. In the in-plane configuration the T1 switch is on while T2 is off; the situation is reversed in the top-down configuration.

**Figure 9:**
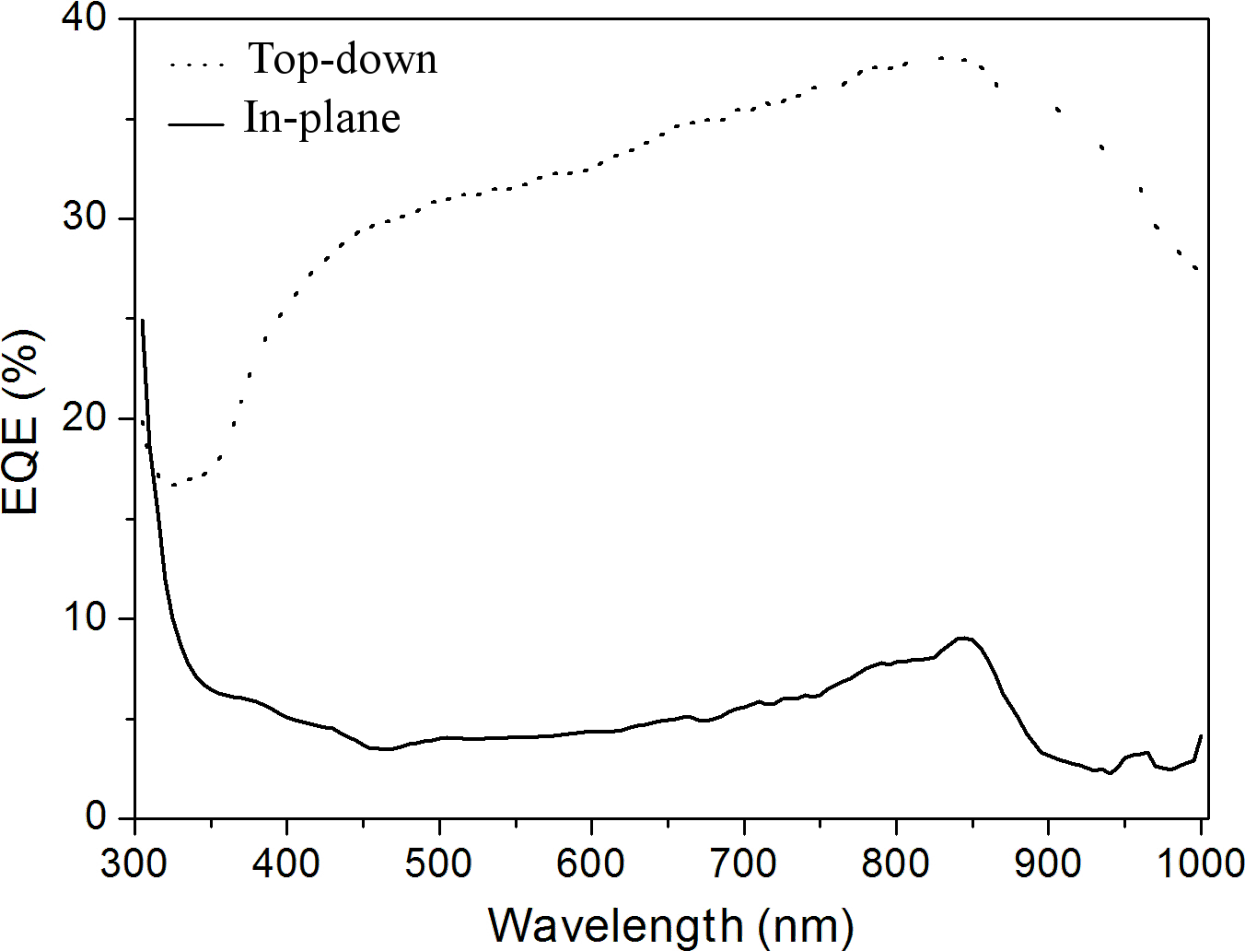
External quantum efficiency (EQE) spectra obtained in the top-down (dotted curve) and in-plane (filled curve) geometries of the device. The maximum value achieved in the case of the top-down configuration is 37% (at 850 nm), while in the planar one it is 10% (at 850 nm). The two line shapes are very different: in the top-down setup the EQE curve is very similar to that of a commercial p–n silicon solar cell, with a low absorption at ultraviolet wavelengths, whereas in the in-plane case, there is a rise that is characteristic of MWCNT absorption.

Figure 9 displays the photoresponse of our device, for both configurations, acquired at null applied voltage and with the light spot (1 × 2 mm^2^) impinging on the MWCNT film. As one can see, the maximum value of EQE achieved in the case of the vertical setup (top-down geometry) is 37%, which is much higher than the 10% obtained in case of the in-plane geometry. Moreover, the EQE line shape is very different in the two cases. While, in top-down geometry, the EQE response clearly resembles the behaviour of a commercial p–n silicon solar cell; in the in-plane architecture there is a further contribution at ultraviolet wavelengths, following the MWCNT optical absorbance. We can assess that in both cases the EQE spectrum is a sum of two contributions, one coming from the MWCNTs and the other from the Si beneath. However, the contribution of the Si is strongly enhanced in the top-down setup, while in the in-plane one the electron–hole pairs generated by Si recombine before reaching the electrodes.

**Figure 10:**
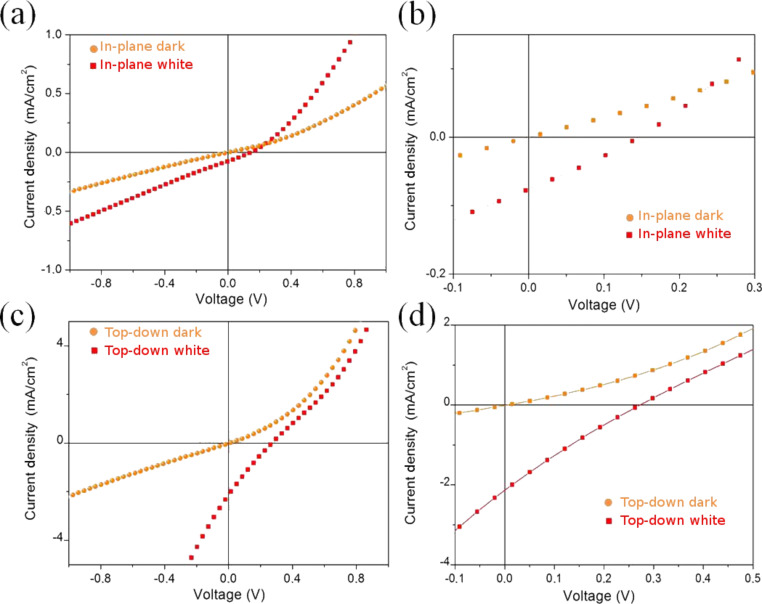
*J*–*V* characteristics acquired in the dark and under illumination by white light. (a) In the in-plane geometry: *V*_oc_ = 0.2 V, *J*_sc_ = 0.09 mA/cm^2^. In the third and fourth quadrant the *J*–*V* curve is almost linear. This could be due to the presence of parasitic resistances, which lower the performance of the device. An inhomogeneous MWCNT film, in which all the MWCNTs are not in good contact with each other, could be the cause of such a high resistance. (b) Detail of (a) in the most meaningful zone, i.e., in the fourth quadrant of the *J*–*V* characteristic. (c) Top-down geometry: *V*_oc_ = 0.25 V, *J*_sc_ = 2.2 mA/cm^2^. Under illumination, the *J*–*V* curve line shape exhibits the behaviour of a double diode: beyond the MWCNT–Si Schottky junction, the second one is formed at the back contact between Si and Al, i.e., a hint of a non-ohmic contact. (d) Detail of (c) in the most meaningful zone, i.e., in the fourth quadrant of the *J*–*V* characteristic.

Strong differences between the two architectures are also evident when we compare the current–voltage curves collected. As reported in Figure 10a and Figure 10b, the open-circuit voltage (*V*_oc_) and the short-circuit density current (*J*_sc_) in the in-plane configuration are markedly lower than those collected in the top-down geometry. The lower value of *J*_sc_ can be justified by the fact that the MWCNTs have essentially a metallic character, thus showing a scarce photovoltaic effect. On the contrary, in the top-down architecture, the main contribution to the photocurrent comes from Si, thus giving a *J*_sc_ value 10^2^ times higher. This particular behaviour can be understood if we consider the position of the electrodes through which the current is collected. In the in-plane geometry, the electrodes are in contact only with the CNT film (and the thick silicon oxide underneath), thus collecting carriers coming mainly from the nanostructures, since the majority of the carriers from the Si recombine during the percolation into the CNT film. On the other hand, in the top-down geometry, we have the two top electrodes contacting the CNT film, while the bottom one is in touch with the silicon substrate. In this way we can collect directly the carriers coming both from the CNTs (top electrodes) and from the Si (bottom).

We notice also that in case of top-down setup the *J*–*V* curve line shape shows a double-diode-like behaviour. This effect is probably due to the presence of a second Schottky junction at the interface between Si and Al at the back of the device, meaning that this contact is not ohmic.

Concerning the solar conversion efficiency, we obtain 0.17% in the top-down setup. This value is in accordance with recently published data obtained for a solar cell based on a MWCNT/Si heterojunction [17]. However, the nearly linear behaviour of the *J*–*V* characteristic in the fourth quadrant under illumination suggests that parasitic resistances are present in the device. For this reason, we think that the performance of our device can be improved by adjustment of different parameters, such as (a) the quality of the contact between the CNTs and the metallic top electrodes; (b) making an ohmic contact at the back of the silicon substrate; and (c) producing a MWCNT film of suitable thickness, thus allowing the photons to reach the heterojunction with the Si substrate.

## Conclusion

In this paper we have shown a facile method to grow CNTs by chemical vapour deposition directly on SS sheet, without the addition of an external catalyst. This implies that there is no need for a additional post-growth purification process. We have highlighted the differences in the electronic properties between MWCNTs and HOPG. In particular, by comparing the ELS spectra we evidenced the presence of electronic structures close to the π-plasmon, which can be assigned to transitions between Van Hove singularities and which explains the ability of CNTs to create electron–hole pairs (excitons) under illumination by light. These features are completely absent in HOPG, for which the π-plasmon peak has a sharp characteristic, and are due to the quasi-one-dimensionality of the CNTs. We have exploited the photovoltaic activity of MWCNTs in a device made of MWCNTs airbrushed onto a Si substrate. We evidenced the formation of a Schottky junction at the interface. EQE spectra and *J*–*V* characteristics were acquired with two different geometries (in-plane and top-down) to study the role played by the excitons generated in the MWCNTs. In the in-plane configuration the main contribution to the photocurrent comes from the MWCNTs, while in the top-down setup the role of silicon dominates, because the excitons generated in the depletion layer are collected before reaching the electrode and recombine. Further improvements should be devoted to enhance the efficiency of the device by improving the quality of the metallic contact, to avoid parasitic additional resistances.

## Experimental

A sheet of AISI 316-SS (30 × 40 mm^2^, from Goodfellow Cambridge Ltd.) was placed on a molybdenum sample holder, acting as a resistive heater, and inserted into the chemical vapour deposition chamber (see Figure 1). After air was removed by a rotary pump, argon gas (12 torr) was inserted. When the desired temperature was reached (750 °C) acetylene gas was introduced (200 sccm) into the chamber to start the CNT growth. After 10 min, the acetylene flow was stopped and argon (500 sccm) was inserted again for 5 min to stop the reaction, while the chamber was pumped off. More details are reported elsewhere [7]. AFM (VEECO multiprobe) characterization was performed in air by using a nonconductive Si tip in tapping mode. After the synthesis process, a piece of SS substrate covered by the as-grown MWCNTs was inserted into an ultrahigh vacuum chamber (base pressure 10^−10^ torr) for Auger and electron energy-loss measurements. An electron gun as excitation source and a semi-imaging electron analyzer MAC2 (Riber Instruments) operating in the constant-pass-energy mode (with a total energy resolution of 1.1 eV) were used. The HOPG sample was exfoliated before introduction to the analysis chamber.

**Figure 11:**
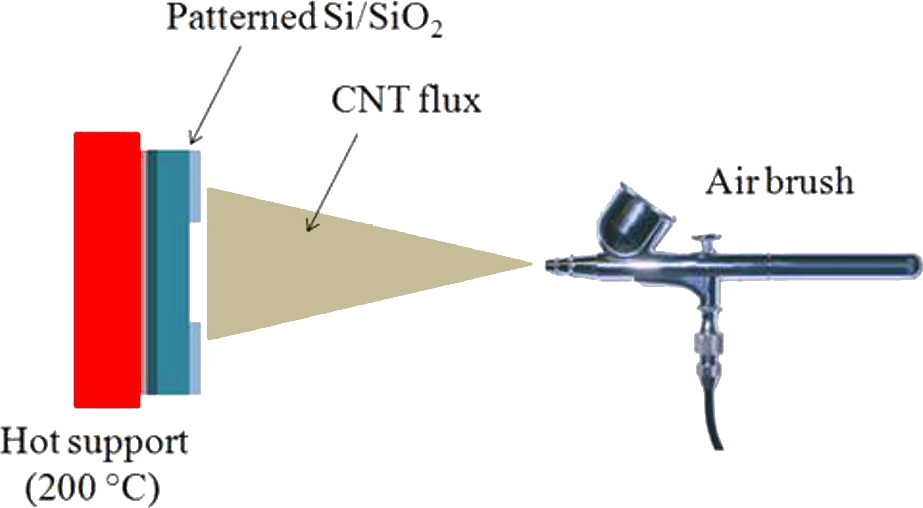
Schematic depiction of the airbrush deposition process. A solution of MWCNTs in isopropyl alcohol was airbrushed onto a Si/SiO_2_ sample. The SiO_2_ steps avoid the formation of a short circuit between the top electrodes and the Si. The Si window was 5 × 5 mm^2^. During airbrushing, the device was mounted on a heatable holder (~200 °C) in order to allow an instantaneous evaporation of the solvent, thus avoiding the formation of droplets.

In order to detach the MWCNTs from the steel substrate after the growth, the sample was sonicated in isopropyl alcohol. The obtained solution was then airbrushed on a patterned n-Si/SiO_2_ substrate (Figure 11). The SiO_2_ steps are needed to prevent a short circuit between the top electrodes and the Si underneath (see Figure 8 and Figure 10). The airbrushed MWCNTs form a quasi-continuous film, which creates the photoactive Schottky heterojunction with the Si. The top electrodes were made of silver paint, while the rear contact was sputtered aluminium. The photocurrent spectra were measured by using an optical setup comprising a xenon lamp equipped with a monochromator, focusing and collecting optics, a reflecting chopper and lock-in electronics. The light spot size was 1 × 2 mm^2^. The photocurrent density, *I*(λ), was measured under illumination as a function of the incident photon wavelength, λ. The incident photon power density was monitored with a calibrated silicon photodiode and data were collected by a lock-in technique. The external quantum efficiency (EQE) is defined as the fraction of the incident photons, *N*_ph_, converted into photocurrent, i.e., the number of the generated electron–hole pairs, *N*_e–h_, multiplied by the electronic charge, *e*. The number of the incident photons is then evaluated in terms of the power density of the Xe lamp, *P*(λ)*,* since *N*_ph_ = λ*P*(λ)/*hc*. Therefore, it results that EQE (%) = electrons/photons = 100 *hcI*(λ)/*e*λ*P*(λ). *I*(λ) was measured by modulating the light by an optical chopper and recovering the amplified current signal (converted to voltage) with a lock-in amplifier locked to the chopper frequency. The lamp power *P*(λ) was measured simultaneously in a similar way, by a calibrated Si photodiode; *h* is the Planck constant, *c* is the speed of light in vacuum and *e* the electron charge. A Keithley 2602A source meter was used to record the *J*–*V* curves both in the dark and under white-light illumination.
